# Identification of a conserved linear B-cell epitope in the M protein of porcine epidemic diarrhea virus

**DOI:** 10.1186/1743-422X-9-225

**Published:** 2012-10-01

**Authors:** Zhibang Zhang, Jianfei Chen, Hongyan Shi, Xiaojin Chen, Da Shi, Li Feng, Bin Yang

**Affiliations:** 1 Division of Swine Infectious Diseases, State Key Laboratory of Veterinary Biotechnology, Harbin Veterinary Research Institute of the Chinese Academy of Agricultural Sciences, No.427 Maduan Street, Nangang District, Harbin, 150001, China; 2 College of Veterinary Medicine, Inner Mongolia Agricultural University, No.306 Zhaowuda Street, Huhhot, 010018, China

## Abstract

**Background:**

The major structural protein of coronaviruses, the membrane (M) protein, can elicit the formation of protective antibodies, but little information is available about the M protein of porcine epidemic diarrhea virus (PEDV). Identification of epitopes on the PEDV M protein will be helpful in the elucidation of the antigenic properties of this protein.

**Results:**

One hybridoma cell line secreting anti-M protein monoclonal antibody (McAb) was generated and designated 4D4. To map the epitopes on the PEDV M protein, a total of 17 partially overlapping fragments covering the C-terminus of M protein were expressed as fusion proteins with a 6×His tag or a GST tag. A linear motif, ^193^TGWAFYVR^200^, was identified by enzyme-linked immunosorbent assay (ELISA) and western blot (WB) analysis using McAb 4D4. The motif ^195^WAFYVR^200^ was the minimal requirement for reactivity, as demonstrated by removing amino acids individually from both ends of the motif ^193^TGWAFYVR^200^. The result of WB analysis showed that the 4D4-defined epitope could be recognized by PEDV-positive serum, but not transmissible gastroenteritis virus (TGEV)-positive serum. Furthermore, this epitope was highly conserved among different PEDV strains, as shown by alignment and comparison of sequences.

**Conclusion:**

A McAb, 4D4, directed against the M protein of PEDV, was obtained, and the 4D4-defined minimal epitope sequence was ^195^WAFYVR^200^. The McAb could serve as a candidate for development of a McAb-based antigen capture ELISA for detection of PEDV. The epitope identified provides a basis for the development of epitope-based differential diagnostic techniques and may be useful in the design of epitope-based vaccines.

## Background

Porcine epidemic diarrhea (PED), which is characterized by severe diarrhea, vomiting and dehydration, is a highly contagious enteric disease of swine and is caused by porcine epidemic diarrhea virus (PEDV) [1]. PED was first reported in England in 1971 [2] and the virus was identified in Belgium and United Kingdom for the first time [3]. Since then it has become prevalent in many swine-raising countries and it is one of the most important viral causes of diarrhea, resulting in heavy economic losses to the swine industry, mainly in European and Asia [4-11]. Although the commercial vaccines are available to prevent and control of disease, damage caused by PEDV infection is serious and continuous.

PEDV, the etiologic agent of PED, belongs to the genus *Alphacoronavirus*, family *Coronaviridae*. It has a genome of single-stranded, positive-sense RNA that is approximately 28 kb in length. The spike protein (S, 180–220kDa), membrane protein (M, 27–32kDa), envelope protein (E, ~7kDa) and nucleocapsid protein (N, 55–58kDa) are the four structural proteins of PEDV [12]. The S protein and M protein are localized on the surface of the virion. The M protein of coronavirus is the most abundant component of the viral envelope. In silico analysis has suggested that the M protein consists of a triple-transmembrane segment flanked by a short amino-terminal domain on the exterior of the virion and a long carboxy-tail located inside the virion. The M protein of coronaviruses is indispensable in the assembly process and budding of virions [13-15]. The immune reaction to the M protein of coronaviruses plays an important role in the induction of protection and in mediating the course of the disease [16,17]. Monoclonal antibodies against the M protein of coronaviruses have virus-neutralizing activity in the presence of complement [18]. The M protein of coronavirus can also stimulate the production of alpha-interferon (α-IFN) [19].

Until now, identification of epitopes on the M protein of PEDV has not been reported. McAb development and epitope mapping of the PEDV M protein will provide the basis for the establishment of diagnostic methods for PEDV infection and also increase understanding of the antigenic structure of the M protein.

## Results

### Production of the fusion protein tM

**Figure 1:**
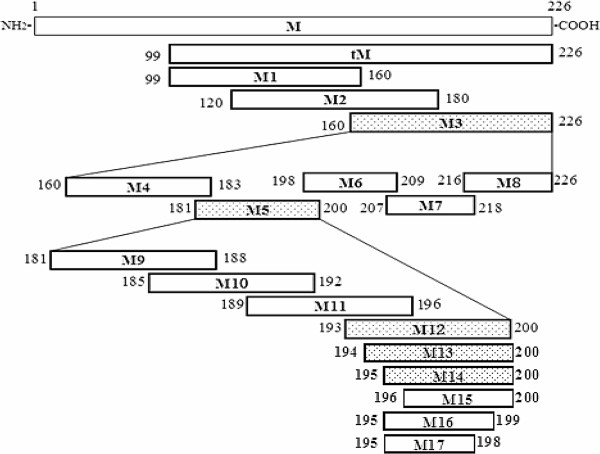
**Schematic diagram of the relative locations of the truncated forms of the M protein of the PEDV CH/SHH/06 strain.** The bars represent the truncated M proteins. The numbers represent the amino acid positions of the M protein. The bars filled with dots represent the peptides that were positive in WB analysis and ELISA with McAb 4D4 and the blank bars represent the peptides that were not recognized by McAb 4D4.

The expression strategy for the fragments of the PEDV M protein is illustrated in Figure 1. In this study, the truncated M (tM) gene, encoding the C-terminus of the M protein (amino acids [aa], 99–226), was expressed as a His_6_-fusion protein and a GST-fusion protein in *E. coli* BL21(DE3), respectively. Both of the fusion proteins, tM-His_6_ and GST-tM, could react with porcine anti-PEDV serum as shown by WB analysis (data not shown), which implies that they had similar antigenicity to the native M protein of PEDV.

### Production and characterization of M protein-specific McAb

The tM-His_6_ protein was used as an immunogen to prepare the McAb, and the GST-tM protein was used as a coating antigen to establish the indirect ELISA for screening the antibody-secreting hybridoma cell lines.

**Figure 2:**
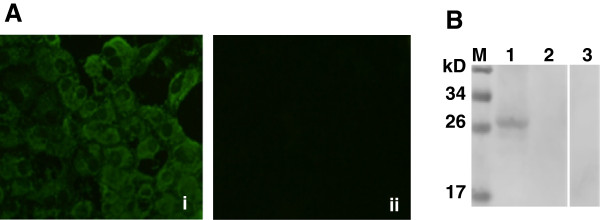
**The McAb 4D4 recognizes PEDV-infected Vero E6 cells and native M protein of PEDV.** (**A**) IFA test of PEDV-infected Vero E6 cells probed with McAb 4D4 (i) and negative serum (ii); (**B**) PEDV particles (lane 1) and TGEV particles (lane 2) were evaluated for reactivity with McAb 4D4 by WB; the PEDV particles (lane 3) incubated with negative serum represent the negative control. M: Protein marker

After cell fusion and screening, one hybridoma clone that secreted McAb specific for the PEDV M protein was isolated and designated 4D4. The isotype of the McAb was IgG2b with *κ* light chain (data not shown). The 4D4 McAb could react with authentic M protein, as shown by immunofluorescent assay (IFA) (Figure 2 A). Moreover, in the WB analysis, the 4D4 McAb recognized a 27-kDa band of the PEDV M protein, while showing no reactivity against the TGEV virion (Figure 2 B). These results demonstrated that the 4D4 McAb recognized specifically the native M protein of PEDV.

### Identification of the epitope by indirect ELISA

To map the antigenic epitope of the PEDV M protein, a set of truncated peptides were expressed in prokaryotes and used to identify the epitope by indirect ELISA. All of the truncated peptides with the expected molecular weights were expressed successfully either with a 6×His tag or with a GST tag (data not shown).

**Figure 3:**
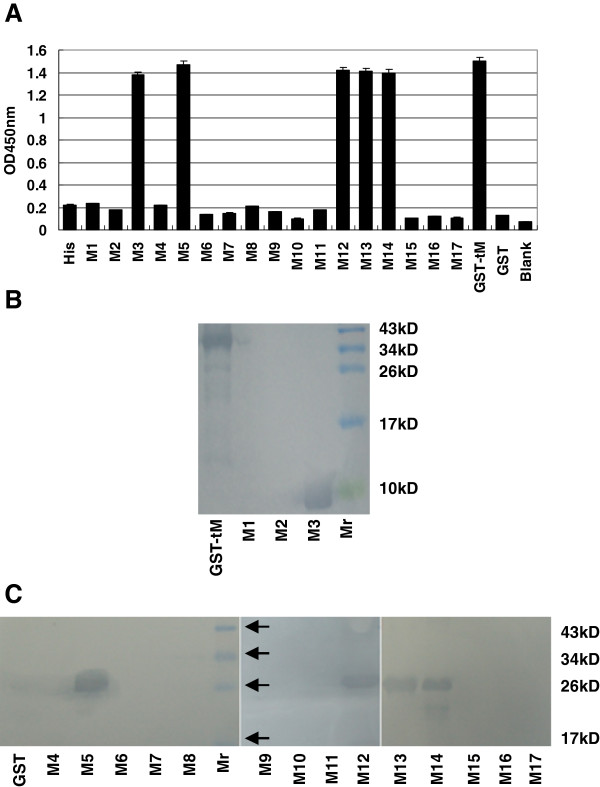
**Precise localization of the McAb 4D4-defined epitope.** The reactivity of McAb 4D4 with different truncated M proteins was determined by ELISA (**A**) and WB (**B** and **C**). The names of the peptides are the same as in Table 1. “His” and “GST”, representing the 6×His tag and the GST tag, were used as negative controls, and the GST-tM protein was used as the positive control. Mr: Protein marker.

First, three overlapping fragments (M1, M2 and M3), covering the C-terminus of the M protein, were expressed as His_6_-fusion peptides, and subjected to ELISA with McAb 4D4 as the primary antibody. As demonstrated by ELISA, M3 showed reactivity with the McAb 4D4 (Figure 3 A). Subsequently, M3 was divided into five GST-fusion fragments (M4–M8) and the five peptides were probed by McAb 4D4. The results of the ELISA indicated that M5 harbored an antigenic epitope (Figure 3 A). To define the epitope more precisely, four 8-mer peptides (M9–M12) spanning the fragment M5 were expressed in a fusion form with GST and detected with the McAb 4D4. The results showed that M12 was recognized by the McAb 4D4, which indicates that the 4D4-specific epitope was ^193^TGWAFYVR^200^ (Figure 3 A).

For fine mapping of the epitope of the M protein, we generated a panel of five shortened peptides (M13–M17) by deleting amino acids individually, at either the amino or the carboxy terminus in sequence, from the peptide M12 (^193^TGWAFYVR^200^). According to the results, the peptides M13 (^194^GWAFYVR^200^) and M14 (^195^WAFYVR^200^) were recognized strongly by the McAb 4D4, whereas M15 (^196^AFYVR^200^), M16 (^195^WAFYV^199^) and M17 (^195^WAFY^198^) failed to show reactivity (Figure 3 A).

### Mapping of the epitope by WB

The results of the ELISA were confirmed further by WB analysis and the results of WB were in accordance with the results of the ELISA (Figure 3 B and C).

Taken together, those results revealed that the minimal linear epitope required for reactivity with the McAb 4D4 was ^195^WAFYVR^200^.

### Reaction of the identified epitope with PEDV-positive serum

**Figure 4:**
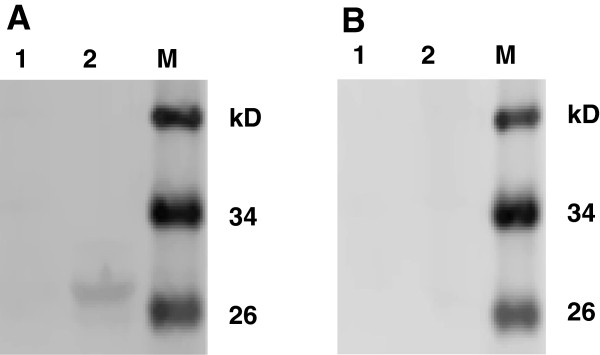
**The epitope M14 could differentiate PEDV-positive serum from TGEV-positive serum.** The WB results for the GST tag (negative control, lane 1) and the defined epitope M14 (lane 2) with PEDV-positive serum (**A**) or TGEV-positive serum (**B**), respectively. M: Protein marker.

As shown by WB analysis, the epitope peptide ^195^WAFYVR^200^, defined by McAb 4D4, could be recognized by PEDV-positive serum from pigs but not TGEV-positive serum, which suggests that this epitope has good reactivity and that porcine anti-PEDV serum contains antibody specific to this epitope (Figure 4 A and B).

### Homology analysis

**Figure 5:**
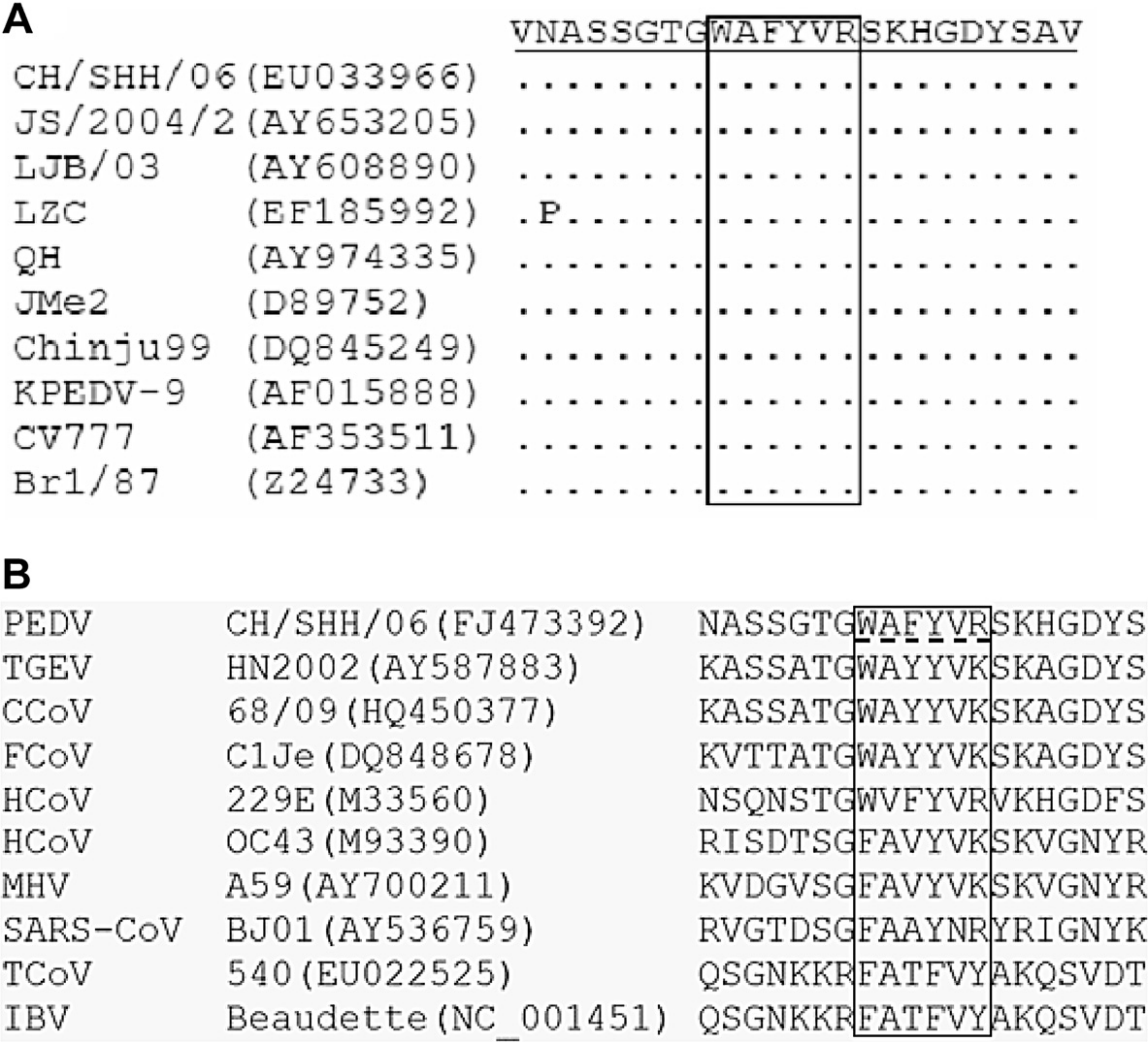
**Alignment of the amino acid sequences of the defined epitope and surrounding region with those of nine PEDV reference strains (A) and nine other coronaviruses (B).** The dots represent residues that match the epitope exactly. The homologous regions of different coronaviruses that correspond to the identified epitope are in the box. Abbreviations of each virus and its strain are listed, and the corresponding GenBank accession numbers are shown in parentheses.

The M gene sequences for residues corresponding to this epitope from 10 different PEDV isolates were used for alignment analysis. The results showed that the epitope ^195^WAFYVR^200^ is totally conserved among these PEDV strains (Figure 5 A), which indicates that the ^195^WAFYVR^200^ sequence represents a conserved epitope on the M protein of PEDV. Analysis of the homologous sequences of the defined epitope from nine different coronaviruses from different groups within the family Coronaviridae demonstrated that the epitope recognized by 4D4 shares low homology among PEDV and other coronaviruses, and that only the residues A^196^, Y^198^ and V^199^ are relatively conserved among all the coronaviruses selected (Figure 5 B).

## Discussion

Mapping epitopes of viral proteins and defining the degree of conservation of identified epitopes may facilitate our understanding of the antigenic structure and virus–antibody interactions, and are useful for clinical application. The epitopes of the PEDV S protein have been reported, and these epitopes were shown to be able to induce the production of virus-neutralizing antibodies [20,21]. However, to date, the epitope on the M protein of PEDV has not been mapped.

Identification of B-cell epitopes on the M protein requires the preparation of McAb. To produce McAb against the M protein, we had tried to express the intact M protein, but this was unsuccessful (data not shown). As a result of unidentified but complex factors, it is very difficult to express the full-length M protein of coronaviruses [22,23]. Finally, we chose to express the truncated C-terminus of the M (tM) protein, and the tM protein was expressed in a fusion form either with a 6×His tag or a GST tag. The results of WB analysis showed that the fusion proteins tM-His_6_ and GST-tM could be recognized by PEDV-positive serum, which indicates that both fusion proteins had good reactivity. In order to obtain more specific McAb, the fusion protein tM-His_6_ was selected as an immunogen to elicit the formation of antibody. The fusion protein GST-tM was chosen to coat the ELISA plates to screen hybridomas, so that the coating antigen bore a different tag from the immunogen. After cell fusion and three cycles of selection, one McAb, designed 4D4, was chosen because of its specific reactivity with the fusion protein GST-tM as well as the native M protein of the PEDV CH/SHH/06 strain.

To map the epitope on the M protein, three overlapping peptides (M1, M2 and M3) covering the tM protein were expressed with a 6×His tag, respectively. Identified by ELISA and WB, the epitope was located at M3, which spans from residues 160 to 226 of the amino acid sequence of the M protein (Figure 3). Nine peptides spanning the identified epitope M3 were expressed in the form of GST-fusion peptides. Further experiments revealed that the epitope was in the M12 region (^193^TGWAFYVR^200^) of the M protein (Figure 3). To define more precisely the core epitope, amino acid residues were removed from the carboxy or amino terminals of the peptide M12 one by one to verify the minimal unit of the epitope that could be recognized by McAb 4D4. As demonstrated in Figure 3, the amino acid residues “^195^W” and “R^200^” were essential for reactivity because deletion of either of them destroyed antibody binding. It is therefore reasonable to deduce that the minimal epitope recognized by McAb 4D4 is ^195^WAFYVR^200^.

Sequence alignment of the amino acids of the identified epitope with those of other PEDV strains and other coronaviruses showed that this epitope was totally conserved across different strains of PEDV, but among different coronaviruses the epitope had only low homology.

To investigate whether the identified epitope ^195^WAFYVR^200^ is specific to PEDV, the peptide was expressed as a GST-fusion protein, and subjected to sodium dodecyl sulfate polyacrylamide gel electrophoresis (SDS-PAGE) and WB analysis. The results demonstrated that the epitope peptide did not react with TGE-positive serum, but it showed reactivity with PEDV-positive serum, indicating that epitope ^195^WAFYVR^200^ is specific for PEDV. Therefore, it was possible to distinguish anti-PEDV antibody from anti-TGEV antibody by employing the epitope as antigen. This will be very helpful in distinguishing PEDV from TGEV infection, because these two diseases have similar clinical and pathological signs, which obscures the differentiation of the two diseases, and diarrhea caused by PEDV and TGEV coinfection occurs frequently in the field.

To our knowledge, the epitopes of coronaviruses that have been identified are mainly focused on the S and N proteins. In the case of the M protein of coronaviruses, Qian *et al.* identified a B-cell antigenic epitope at the N-terminus of the severe acute respiratory syndrome coronavirus (SARS–CoV) M protein [24], and Xing *et al.* reported a linear B-cell epitope in the M protein of avian infectious bronchitis coronavirus (IBV) [25]. The epitope of the IBV M protein identified by Xing *et al.* was ^199^FATFVYAK^206^. Comparative analysis of the epitope ^199^FATFVYAK^206^ with the corresponding regions of other coronaviruses revealed that the homologous region of the PEDV M protein was ^195^WAFYVRSK^202^[25]. The epitope of the M protein of PEDV defined in this study was ^195^WAFYVR^200^, and by comparison the homologous region of the IBV M protein was ^199^FATFVY^204^. So, interestingly, the results of our study are consistent with findings regarding the IBV M protein.

## Conclusion

In the current study, we generated a McAb, 4D4, and mapped the epitope on the M protein of PEDV using the McAb. The linear B-cell epitope identified, ^195^WAFYVR^200^, was highly conserved among different PEDV isolates. Furthermore, this epitope could differentiate PEDV-positive serum from TGEV-positive serum. These data could deepen understanding of the antigenic structure of the M protein. The information regarding the epitope obtained in this study could be used to establish a differential diagnostic test and may be helpful in the design of multi-epitope vaccines.

## Methods

### Cell lines, viruses and serum specimens

Myeloma cell line SP2/0 and Vero E6 cells were cultured in Dulbecco’s modified Eagle’s medium (DMEM, Hyclone) in humidified 5% CO_2_ atmosphere at 37°C. All culture media were supplemented with 10% heat-inactivated fetal bovine serum (PAA, Somerset, UK) and antibiotics (0.1mg/ml of streptomycin and 100IU/ml of penicillin). The PEDV CH/SHH/06 strain (EU033966) and TGEV attenuated strain H (EU074218) were maintained in our laboratory. Hyperimmune serum against PEDV and TGEV were obtained from pigs that had been inoculated with purified PEDV or TGEV.

### Expression of the truncated form of the PEDV M gene

A truncated form, comprising 387 bp at the C-terminus of the M gene of PEDV CH/SHH/06 strain, was cloned. The sequences of the primers used for amplification of the gene in this study are shown in Table 1.

**Table 1:** Sequences of the primers used in this study

**Peptides**	**Primer sequences (5’→3’)**	**Position**	**Size of amplicon**
tM-His_6_	F: ATACATATGAGCATTCGGTTGTGGCG	295–678	384bp
	R: CGCCTCGAGGACTAAATGAAGCACT		
	F: TAAGGATCCAGCATTCGGTTGTGGCGCAG		
GST-tM	R: CGGCTCGAGGACTAAATGAAGCACTTTCTCAC	295–678	384bp
	F: TATCATATGAGCATTCGGTTGTGG		
M1	R: TATCTCGAGTACGCCAGTAGCAAC	295–480	186bp
	F: GCGCATATGCTCACTACTTCTGTGAT		
M2	R: GCGCTCGAGATAGACAATTGTTGTAG	358–540	183bp
	F: GCACATATGGTACAGGTAAGTCAAT		
M3	R: CTACTCGAGGACTAAATGAAGCACT	478–678	201bp
	F: *gatcc*GTACAGGTAAGTCAATTACCTAATTTCGTCACAGTCGCCA		
M4	AGGCCACTACAACAATTGTCTATGGACGTGTT**taa***c*	478-549	72bp
	R: *tcgag***tta**AACACGTCCATAGACAATTGTTGTAGTGGCCTTGGCGA		
	CTGTGACGAAATTAGGTAATTGACTTACCTGTAC*g*		
	F: *gatcc*GGACGTGTTGGTCGTTCAGTCAATGCTTCATCTGGCACTG		
M5	GTTGGGCTTTCTATGTCCGG**taa***c*	541–600	60bp
	R: *tcgag***tta**CCGGACATAGAAAGCCCAACCAGTGCCAGATGAAGCA		
	TTGACTGAACGACCAACACGTCC*g*		
M6	F: *gatcc*TCAGCTGTGAGTAATCCGAGTGCGGTTCTCACAGAT**taa***c*	592–627	36bp
	R: *tcgag***tta**CACAGCTGAGTAGTCGCCGTGTTTTGACCGGACATA*g*		
M7	F: *gatcc*TCAGCTGTGAGTAATCCGAGTGCGGTTCTCACAGAT**taa***c*	619–654	36bp
	R: *tcgag***tta**ATCTGTGAGAACCGCACTCGGATTACTCACAGCTGA*g* F: *gatcc*CTCACAGATAGTGAGAAAGTGCTTCATTTAGTC**taa***c*		
M8	R: *tcgag***tta**GACTAAATGAAGCACTTTCTCACTATCTGTGAG*g*	646–678	33bp
M9	F: *gatcc*GGACGTGTTGGTCGTTCAGTCAAT**taa***c*	541–564	24bp
	R: *tcgag***tta**ATTGACTGAACGACCAACACGTCC*g*		
M10	F: *gatcc*CGTTCAGTCAATGCTTCATCTGGC**taa***c*	553–576	24bp
	R: *tcgag***tta**ATTGACTGAACGACCAACACGTCC*g*		
M11	F: *gatcc*GCTTCATCTGGCACTGGTTGGGCT**taa***c*	565–588	24bp
	R: *tcgag***tta**AGCCCAACCAGTGCCAGATGAAGC*g*		
M12	F: *gatcc*ACTGGTTGGGCTTTCTATGTCCGG**taa***c*	577–600	24bp
	R: *tcgag***tta**CCGGACATAGAAAGCCCAACCAGT*g*		
M13	F: *gatcc*GGTTGGGCTTTCTATGTCCGG**taa***c*	580–600	21bp
	R: *tcgag***tta**CCGGACATAGAAAGCCCAACC*g*		
M14	F: *gatcc*TGGGCTTTCTATGTCCGG**taa***c*	583–600	18bp
	R: *tcgag***tta**CCGGACATAGAAAGCCCA*g*		
	F003A *gatcc*GCTTTCTATGTCCGG**taa***c*		
M15	R: *tcgag***tta**CCGGACATAGAAAGC*g*	586–600	15bp
	R: *tcgag***tta**GACATAGAAAGCCCA*g*		
M16	F: *gatcc*TGGGCTTTCTAT**taa***c*	583–597	15bp
	R: *tcgag***tta**GACATAGAAAGCCCA*g*		
M17	F: *gatcc*TGGGCTTTCTAT**taa***c*	583–594	12bp
	R: *tcgag***tta**ATAGAAAGCCCA*g*		

The introduced restriction enzyme sites (NdeI and XhoI or BamHI and XhoI) are underlined. At the 5’ and 3’ terminal of each forward strand there is a sequence “gatcc” and “taac” (in italics), respectively. At the 3’ and 5’ terminal of each reverse strand there is a sequence “g” and “tcgagtta” (in italics), respectively. When the forward and reverse oligonucleotides annealed they form a cohesive BamHI site or NdeI at the 5’ terminus and a cohesive XhoI site at the 3’ terminus. The bases “taa” and “tta” (in bold) were introduced into each pair of oligonucleotides to form a stop codon.

The amplified truncated form of the M gene (tM, 387bp), encoding the C-terminus of M protein (aa, 99–226), was cloned into prokaryotic expression vectors pET-30a (Novagen, USA) and pGEX-6p-1 (Pharmacia, Belgium), respectively. The inserts in the recombinant plasmids were sequenced and the confirmed plasmids were transformed into *E*. *coli* BL21(DE3) and induced by Isopropyl β-D-1-thiogalactopyranoside (IPTG). The expressed fusion proteins were analyzed with SDS-PAGE and detected by staining with Coomassie blue. For preparation of purified proteins, inclusion body proteins were separated by SDS-PAGE, the proteins of interest were excised, and the gel slices were crushed and added to an appropriate volume of sterilized phosphate buffered saline (PBS). After several cycles of freeze–thawing, the fusion proteins were dissolved in PBS, and the PBS was separated from the solid gel by centrifugation. The purity of the fusion proteins generated was analyzed by SDS-PAGE, and the purified proteins were used for ELISA and WB analysis.

### Preparation and characterization of McAb against the tM protein

Female six-week-old BALB/c mice were primed subcutaneously with purified tM-His_6_ protein emulsified with an equal volume of Freund’s complete adjuvant (Sigma, USA). Two booster immunizations were given at two-week intervals with the fusion protein tM-His_6_ in Freund’s incomplete adjuvant. A final immunization containing purified tM-His_6_ protein without adjuvant was injected intraperitoneally. Three days after the final injection, the mice were euthanized and their splenocytes were harvested and fused with SP2/0 myeloma cells using polyethylene glycol 1450 (PEG1450, Sigma, USA). The hybridoma cells were seeded into 96-well plates and selected in hypoxanthine–aminopterin–thymidine (HAT) medium and hypoxanthine–thymidine (HT) medium in sequence. Cell culture supernatants of surviving clones were screened for antibody reactivity and specificity by ELISA. The positive hybridoma cell clones were subcloned thrice by limiting dilution. Ascites fluid was produced in separate primed BAB/c mice. The class and subclass of the McAb was determined using a SBA Clonotyping^TM^ System/HRP (Southern Biotechnology Associates, Inc., Birmingham, AL35260, USA).

Animal care and all procedures were performed in accordance with animal ethics guidelines and approved protocols. The animal experiment was approved by Harbin Veterinary Research Institute. The animal Ethics Committee approval number is Heilongjiang-SYXK-2006-032.

Immunofluorescence assay was performed to assess whether the McAb 4D4 could recognize the native M protein. Vero E6 cells were infected with the CH/SHH/06 strain of PEDV and incubated at 37°C for 24 h. The cells were fixed with cold methanol for 10 min and then probed with McAb 4D4 and negative normal mouse serum for 1 h at 37°C. A fluorescein isothiocyanate (FITC)-conjugated goat anti-mouse IgG was used as a secondary antibody, and bound antibodies were visualized under a fluorescence microscope.

The specificity and reactivity of the McAb was determined by WB analysis using purified virus particles of the CH/SHH/06 strain of PEDV and the attenuated H strain of TGEV.

### Expression of the truncated forms of the tM gene

To map the epitope of the M protein, a panel of 17 partially overlapping peptides (M1–M17) spanning the tM protein, as shown in Figure 1, were expressed successively. For peptides M1–M3, three pairs of primers were designed to amplify the genes of the fragments, and peptides M1–M3 were expressed with a 6×His tag, respectively. For each peptide M4–M17, a pair of oligonucleotide strands was synthesized. Each pair of oligonucleotide strands was annealed, and the resultant double-stranded DNA contained a BamHI and a XhoI cohesive terminus at the 5' and 3' ends. The annealed fragments were cloned into the expression vector pGEX-6p-1 and expressed as GST fusion proteins.

The recombinant plasmids thus constructed were sequenced and the confirmed recombinant plasmids were transformed into *E*. *coli* BL21(DE3). A series of fusion peptides were induced by IPTG and stained with Coomassie blue after SDS-PAGE. Purification of the short fusion peptides was carried out as mentioned above.

### Identification of the epitope by indirect ELISA

Ninety-six-well microtiter plates were coated with the purified proteins in 0.1 M carbonate buffer (pH 9.6) at 4°C overnight and blocked with 5% skim milk for 1 h. After washing three times with PBST, 100 μl of McAb was added to the wells and incubated at 37°C for 1 h. Subsequently, the plates were washed thrice and incubated with horseradish peroxidase (HRP)-conjugated goat anti-mouse IgG at 37°C for 1 h. The color was developed and the reaction was terminated with 2 M H_2_SO_4_. The absorbance was measured at 450 nm. All assays were performed in triplicate and the results depict the average of the three values.

### Mapping of the epitope by WB

The reactivity of the McAb with different truncated M proteins was determined further by WB. Purified truncated proteins were subjected to electrophoresis using 12% SDS-PAGE and transferred onto a nitrocellulose (NC) membrane. Non-specific antibody binding sites were blocked with 5% skim milk in PBS overnight at 4°C. The membranes were incubated with primary antibody at 37°C for 1 h and then washed three times with PBST. Next, the stripes were probed with HRP-conjugated goat anti-mouse IgG (Sigma, USA) for 1 h at 37°C. After three final wash steps, the color was developed using 3,3'-diaminobenzidine (DAB) substrate and was stopped finally by rinsing in deionized water.

### Detection of the reactivity and specificity of the epitope defined by McAb 4D4

To investigate the reactivity and specificity of the epitope peptide defined by McAb 4D4, the peptide M14 “GST-WAFYVR” was purified and subjected to WB to investigate its reactivity with PEDV-positive serum, TGEV-positive serum and pre-immune serum from pigs, respectively. The WB was performed as described above, and HRP-conjugated goat anti-swine IgG was used as the secondary antibody.

### Homology analysis

To analyze the conservation of the identified epitope among PEDV reference strains, the epitope sequence and flanking sequences of the M protein were compared with those of nine other selected PEDV strains using the DNAMAN software (Lynnon BioSoft Inc., USA). Alignment analysis was also performed between the defined epitope and the corresponding regions of other associated coronavirus strains using the DNASTAR Larsergene program (Windows version; DNASTAR Inc., Madison, WI, USA).

## Competing interests

The authors declare that they have no competing interests.

## Authors’ contributions

LF designed the experiment. ZZ carried out most of the experiments and wrote the manuscript. XC, BY and DS prepared the McAb and analyzed the data from the ELISA. HS participated in some of the experiments. JC and LF revised the manuscript. All the authors read and approved the final manuscript.
